# Efficacy of laparoscopic sleeve gastrectomy for patient with morbid obesity and type 1 diabetes mellitus: a case report

**DOI:** 10.1186/s40792-020-00989-5

**Published:** 2021-01-06

**Authors:** Hidetaka Ichikawa, Hirofumi Imoto, Naoki Tanaka, Hiroaki Musha, Shojiro Sawada, Takeshi Naitoh, Takashi Kamei, Michiaki Unno

**Affiliations:** 1 Department of Surgery, Tohoku University Graduate School of Medicine, Seiryo-machi, Aoba-ku, Sendai, 980-8574 Japan; 2 Department of Metabolism and Diabetes, Tohoku University Graduate School of Medicine, 1-1, Seiryo-machi, Aoba-ku, Sendai, 980-8574 Japan; 3 Department of Colorectal Surgery, Kitasato University School of Medicine, 1-15-1, Kitasato, Minami-ku, Sagamihara, 252-0374 Japan; 4 Department of Diabetes and Metabolism, Tohoku Medical and Pharmaceutical University, Fukumuro, Miyagino-ku, Sendai, 983-8512 Japan

**Keywords:** Type 1 diabetes mellitus, Morbid obesity, Laparoscopic sleeve gastrectomy, Metabolic surgery

## Abstract

**Background:**

Bariatric surgery is effective for the treatment of patients with morbid obesity and type 2 diabetes mellitus (T2DM), for body weight loss and glycemic control. However, in Japan, there has been no previous report of the effectiveness bariatric surgery in a case of morbid obesity associated with acute onset type 1 diabetes mellitus (T1DM), in which pancreatic β-cells were destroyed and endogenous insulin was depleted.

**Case presentation:**

A 36-year-old woman with morbid obesity and T1DM, diagnosed when she was 6 years, was admitted for bariatric surgery. At her first consultation, she had a body weight of 106.7 kg and a body mass index of 42.2 kg/m^2^. Her HbA1c level was 9.0%, with a required daily insulin dose of 75 units. She underwent laparoscopic sleeve gastrectomy. At 1 year after surgery, her body weight had decreased to 81.0 kg and her body mass index to 32.2 kg/m^2^. In addition, her daily required dose of insulin had decreased to 24 units, with an improvement in her HbA1c level to 7.7%.

**Conclusions:**

Although further evidence needs to be accumulated, including long-term outcomes, laparoscopic sleeve gastrectomy may provide an effective treatment for patients with morbid obesity and T1DM for body weight loss, improvement in HbA1c level, and insulin dose reduction.

## Background

Bariatric surgery for morbid obesity is widely performed around the world [1], with demonstrated effectiveness in improving type 2 diabetes mellitus (T2DM) [2, 3]. Furthermore, the improvement effect on glycemic control after this surgery are observed prior to body weight loss, with the metabolic effects being markedly greater than can be explained by the loss of body weight alone. In recent years, "Metabolic Surgery" has been introduced as a new concept. However, it is not clear how this concept might apply differently to type 1 diabetes mellitus (T1DM) compared to T2DM.

T1DM is a disease in which pancreatic β-cells are destroyed and insulin secretion becomes impaired. Almost in the same way as T2DM, failure of glycemic control in the chronic phase of T1DM can lead to microangiopathy (retinopathy, nephropathy, neuropathy) and macroangiopathy (atherosclerosis), which can worsen the prognostic outcomes of patients. The main treatment for T1DM is insulin therapy. In recent years, the number of patients with morbid obesity and T1DM has increased. Bae et al. reported that analyzed electronic health records in the United States estimated that 47.8% of patients with T1DM are obese [4]. Several studies on the usefulness of bariatric surgery for these cases having emerged [5–9]. In Japan, only two studies have described the effect of bariatric surgery on slowly progressive insulin-dependent diabetes mellitus (SPIDDM), which is included in T1DM [10, 11], and no studies on bariatric surgery for patients with severe obesity and T1DM with insulin secretion deficiency. In this case report, we describe the effectiveness of laparoscopic sleeve gastrectomy (LSG), by reducing the size of the stomach, in a patient with morbid obesity and T1DM, without endogenous insulin, achieving weight loss, a marked reduction in insulin requirement, and improved glycemic control.

## Case presentation

A 36-year-old Japanese female was referred to our hospital with morbid obesity and T1DM. She was diagnosed with T1DM at the age of 6 years, thereafter, treatment with multiple daily insulin was started. By the age of 20 years, she had a body weight of 70 kg, increasing to > 100 kg at the age of 34 years. Her required daily dose of insulin increased as a function of her body weight. At her initial assessment, she required 45 units of insulin aspart and 30 units of insulin glargine per day. Although a temporary weight loss and reduction in daily insulin dose was achieved with an in-hospital treatment, her weight rebounded shortly after discharge and the patient experienced difficulty in controlling her body weight. The patient expressed her intention for surgical treatment for weight loss, and she was referred to our department.

**Fig. 1:**
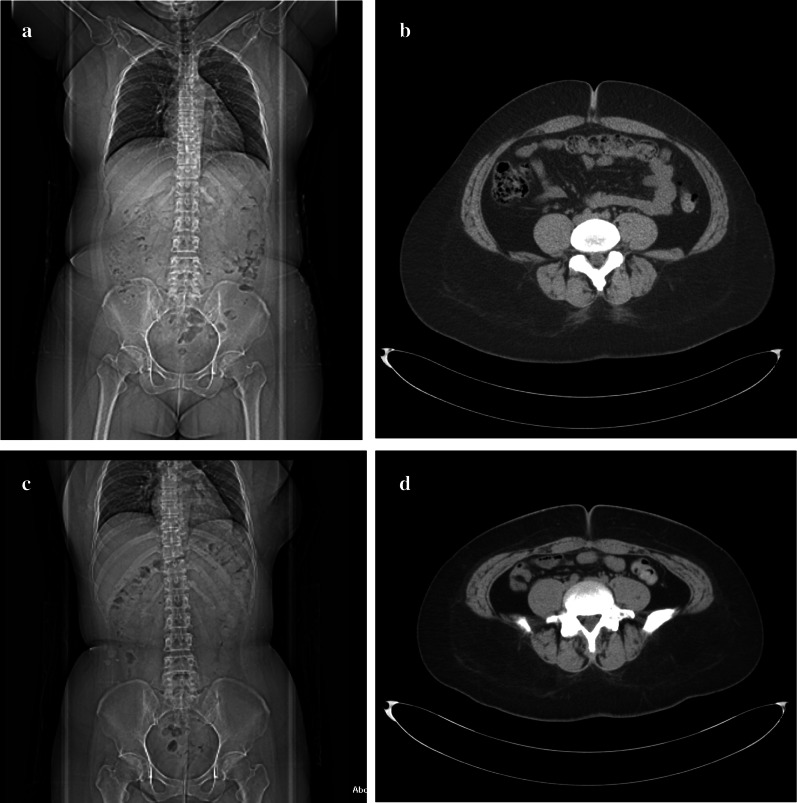
Computed tomography images. **a** Overall image before surgery, showing **b** a preoperative visceral fat area of 162.6 cm^2^ and subcutaneous fat area of 527.9 cm^2^. **c** Overall image, 1-year after the surgical procedure, showing a decrease in **d** the visceral fat area to 44.8 cm^2^ and the subcutaneous fat area to 408.8 cm^2^

At the time of admission, her height was 159 cm and her weight 106.7 kg, BMI of 42.2 kg/m^2^. Blood analyses indicated HbA1c of 9.0%, and blood C-peptide levels were undetectable (< 0.01 ng/mL), suggesting her insulin secretion capacity was completely depleted. With medication, her blood lipid levels were within normal range. On computed tomography (CT) examination, the calculated visceral fat area was 162.6 cm^2^, with a subcutaneous fat area of 527.9 cm^2^, measured at level of the umbilicus (Fig. 1a, b). Upper gastrointestinal endoscopy revealed no abnormalities in the esophagus, stomach, or duodenum.

To prevent complications associated with rapid postoperative blood glucose improvement, she was admitted to our hospital 2 weeks before operation for strict glycemic control, dietary restrictions, and exercise therapy. As a result, preoperative HbA1c was reduced to 7.8% and body weight was reduced to 101.1 kg.

**Fig. 2:**
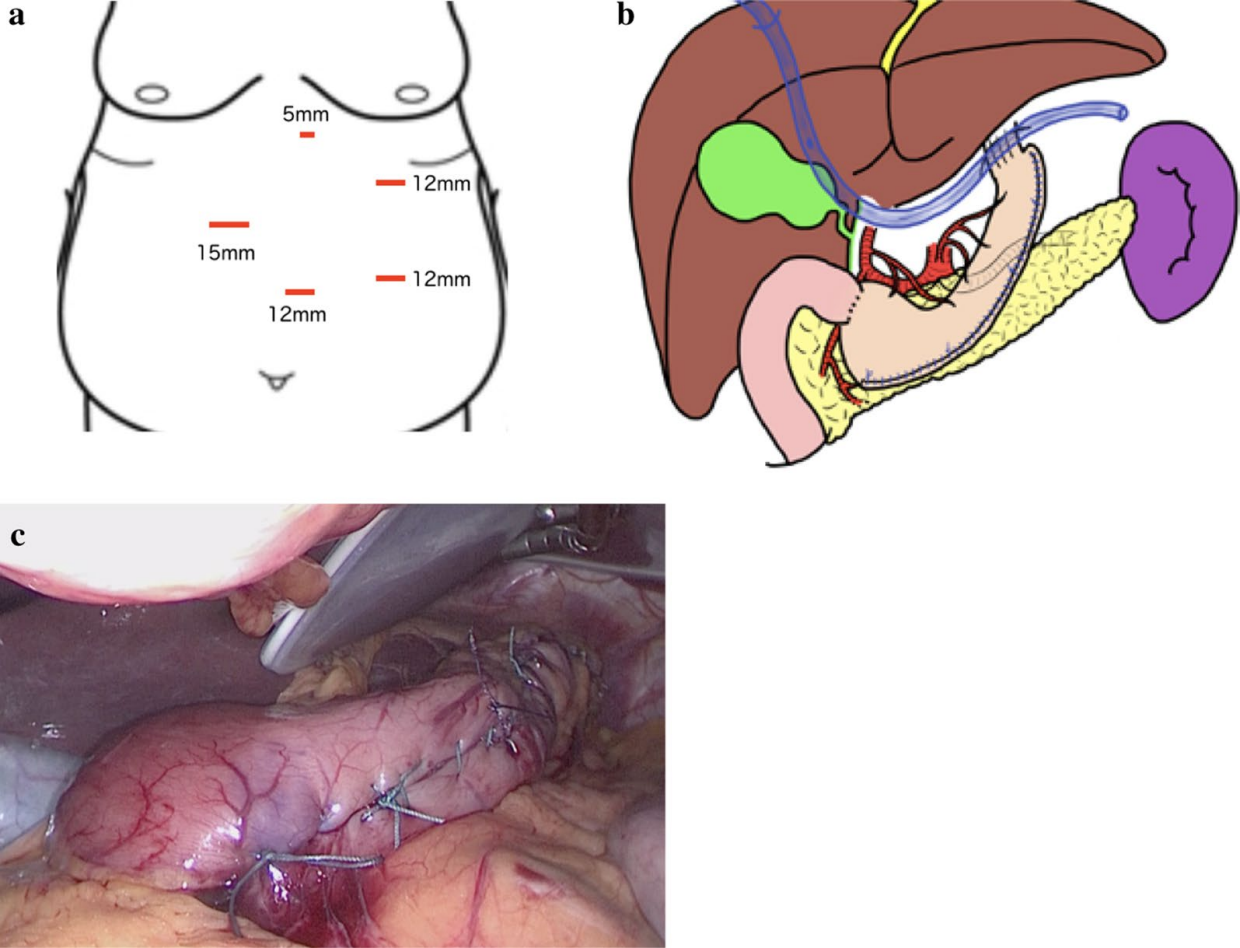
Surgical schema and gastric tube. **a** Schema of skin incisions (red lines), with the layout and size of ports shown. **b** Surgical schema, showing a drain placed below the left diaphragm. **c** Intraoperative photograph, with the complete gastric tube shown

We performed a laparoscopic sleeve gastrectomy (LSG) [12], using five ports,, as shown in Fig. 2a. The blood vessel along the wall of the greater curvature of the stomach was first dissected. We then inserted a 36 Fr (12 mm) bougie into the stomach and resected the greater curvature of the stomach, from a point, on the oral side, 4 cm from the pylorus to the His angle, using a linear stapler. The staple line was reinforced with continuous seromuscular sutures using non-absorbable stitches (Fig. 2b, c).

After the operation, a unit of insulin aspart was mixed with 5 g of glucose contained in the infusion solution and sliding scale insulin was added as needed. From postoperative day 2, insulin glargine was administered. Sliding scale insulin was added depending on fasting blood sugar level and oral intake and her daily insulin dose was determined accordingly.

**Fig. 3:**
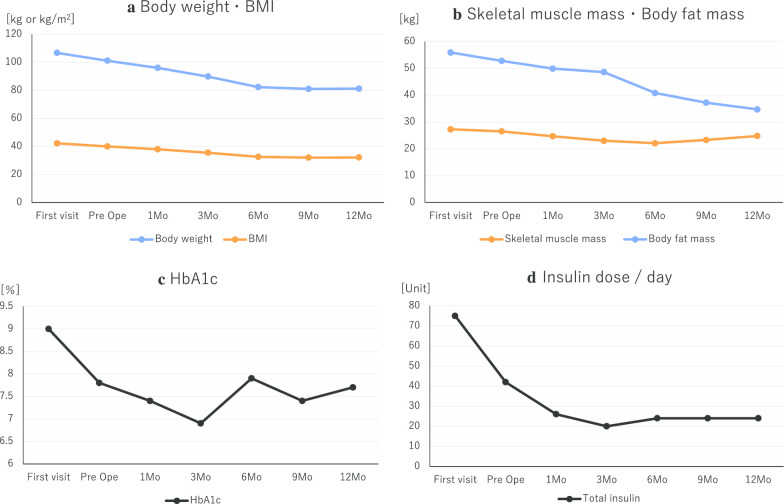
Postoperative changes. The change, from preoperative to 12 months postoperatively, in **a** body weight, body mass index (BMI); **b** skeletal muscle mass and body fat mass; **c** HbA1c; and **d** insulin dose/day. At 1-year after the procedure, the patient’s body weight had decreased to 81 kg and her BMI to 32.2 kg/m^2^, mainly due to a decrease in body fat mass, with the skeletal muscle mass being maintained. The HbA1c level improved to 7.7%, and the daily insulin dose required reduced to 24 units

There were no postoperative complications, including severe hypoglycemic episodes. One year after the procedure, her body weight had decreased to 81.0 kg, with a BMI of 32.2 kg/m^2^, with this decrease being mainly due to a decrease in the body fat mass. Her HbA1c level improved to 7.7%, and her daily required insulin dose had been reduced to 24 units (10 units of insulin aspart and 14 units of insulin glargine per day: Fig. 3a–d). On abdominal CT images, the visceral fat area, measured at level of the umbilicus, was 44.8 cm^2^, with a subcutaneous fat area of 408.8 cm^2^ (Fig. 1c, d). Therefore, there was a marked decrease in both visceral and subcutaneous fat.

## Discussion

According to the International Federation for the Surgery of Obesity and Metabolic Disorders (IFSO), about 340,000 bariatric surgeries were performed, worldwide, in 2008, with this number doubling by 2016 to over 680,000, most of which were performed laparoscopically [1]. In Japan, only LSG has been covered by national insurance since 2014, with the number of LSG procedures performed having increased every year since then. It is well known that bariatric surgery is effective for weight-loss effect, as well as improving T2DM for a prolonged period after surgery [2, 3] and lowering the risk for obesity-related diseases, such as cardiovascular disorders [13]. However, there are few reports of the therapeutic effect of LSG in patients with T1DM, and it has not yet been elucidated and remains controversial.

T1DM is caused by the destruction of pancreatic β cells due to the interaction between genetic factors, environmental factors, and autoimmune mechanisms. According to a survey of the incidence of childhood T1DM in countries around the world, the age-adjusted incidence is high in Europe and in the United States, and low in Japan, at about 2.37 per 10,000 individuals [14, 15]. T1DM presents with a variety of clinical features and is classified into three types, according to the mode of onset: typical acute-onset type; SPIDDM, which presents with T2DM pathology at the time of diagnosis and endogenous insulin secretion gradually decreases, with progression to insulin dependence; and fulminant type, characterized by a rapid destruction of pancreatic β cells, leading to severe hyperglycemia which can sometimes be fatal. For all three types of T1DM, insulin therapy is the main treatment. Poor glycemic control over a prolonged period of time causes microangiopathy (retinopathy, nephropathy, neuropathy) and macroangiopathy (atherosclerosis), as with T2DM, with a significant negative impact on patient prognosis.

The cause of poor glycemic control in T2DM is mainly due to obesity and insulin resistance. This is important to note as the rate of obesity among adults with T1DM has been increasing. In recent years, the concept of “double diabetes” [16, 17] has been proposed. This is a new expression of the disease in children and adolescents, with the characteristics of a mixture of the two types of diabetes as patients with T1DM diagnosed in infancy acquire the T2DM factor from adolescence to adulthood. This mixed presentation induces obesity and insulin resistance, which leads to poor glycemic control and an increase in the amount of required daily insulin.

There have been a few reports on the efficacy of bariatric surgery in patients with morbid obesity and T1DM [5–9]. The systematic review by Chow et al. summarizes the outcomes of bariatric surgery in 86 patients with T1DM [5]. Before surgery, the average BMI was 42.5 ± 2.65 kg/m^2^, with an average HbA1c level of 8.46 ± 0.78% and average required insulin dose of 98 ± 26 IU/day. One year after surgery, the BMI had decreased to 29.55 ± 1.76 kg/m^2^, the HbA1c level to 7.95 ± 0.55%, and the required insulin dose to 36 ± 15 IU/day. Furthermore, the risk for obesity-related diseases had also been reduced after surgery [8, 9].

In Japan, bariatric surgery for T1DM has been reported only for cases of SPIDDM [10, 11]; in these cases, it was possible to reduce or discontinue insulin preparations and oral glycemic drugs after surgery. As an explanatory mechanism, the authors proposed that postoperative weight loss improved insulin resistance, resulting in a protective effect on residual pancreatic β cells. However, there has been no previous report of the effectiveness bariatric surgery in a case of morbid obesity associated with typical acute-onset T1DM, in which pancreatic β-cells were destroyed and endogenous insulin was depleted. This is the first case report of typical acute-onset T1DM with endogenous insulin depletion in Japan. In this case, weight loss and improved glycemic control were achieved in the postoperative follow up period, especially the amount of daily insulin requirement was decreased more dramatically than the weight reduction. This suggests that the observed metabolic effect is not just as a result of the restrictive effect of the surgery or due to the loss in body weight alone. In considering this mechanism of improvement, the concept of “double diabetes” [16, 17] is thought to be useful. In other words, it is presumed that the effectiveness of the bariatric surgery is mediated by an improvement in the T2DM factor among patients with double diabetes. Ashrafian et al. reported that after bariatric surgery, β cell dysfunction persisted and, thus, patient still required baseline insulin therapy, although the overall insulin requirement was reduced [7]. Incretin hormones may also play an important role. In T2DM, change in the dynamics of incretin hormone secretion, such as glucagon-like peptide-1 (GLP-1), after gastric bypass surgery, contributes to the postoperative improvement in glycemic control [18]. It is plausible that incretin hormones may also contribute to the improvement of glucose metabolism in patients with T1DM after bariatric surgery through an inhibition of glucagon secretion via α cells, even in patients without residual β cells [19]. However, the underlying mechanisms remain to be elucidated.

Our case shows the possible usefulness of bariatric surgery for the treatment of patients with morbid obesity and T1DM, without endogenous insulin, to achieve postoperative weight loss and to improve glycemic control 1 year after surgery. On the other hand, Vilarrasa et al. described that HbA1c, which had improved in the first year after surgery, returned to the preoperative baseline after 5 years [6]; therefore, our case also requires long-term strict follow-up. Accumulation of more cases and evaluation of long-term results are warranted to improve our understanding of the role of bariatric surgery for patients with obesity and T1DM.

## Conclusion

In the short term, LSG would provide an effective treatment strategy for patients with morbid obesity and T1DM to achieve body weight loss, improve HbA1c level, and reduce the required daily insulin dose.

## Data Availability

The dataset supporting the conclusions of this article is included within the article.

## Abbreviations

T2DM: Type 2 diabetes mellitus
T1DM: Type 1 diabetes mellitus
LSG: Laparoscopic sleeve gastrectomy
CT: Computed tomography
SPIDDM: Slow progressive insulin dependent diabetes mellitus
GLP-1: Glucagon-like peptide-1
